# LGBTQ+ Healthcare Teaching in UK Medical Schools: An Investigation into Medical Students’ Understanding and Preparedness for Practice

**DOI:** 10.1177/23821205231164893

**Published:** 2023-03-27

**Authors:** Alice Barber, Alexander Flach, Jack Bonnington, Emily M Pattinson

**Affiliations:** 1 4468 School of Medicine, University of Leeds, Leeds, UK; 2 Newcastle University, Newcastle upon Tyne, UK

**Keywords:** LGBTQ+, medical curriculum, curriculum change, preparedness for practise

## Abstract

**OBJECTIVES:**

Lesbian, gay, bisexual, trans* and queer/questioning + (LGBTQ+) healthcare teaching within UK medical schools is currently lacking, potentially impacting on patients’ confidence in health services and ability to access care. The current study conducted a multi-site analysis aiming to investigate medical students' perceptions towards the teaching of LGBTQ+ healthcare in UK medical schools, as well as to gain a greater understanding of medical students’ level of knowledge of LGBTQ+ healthcare, and preparedness for working with LGBTQ+ patients.

**METHODS:**

Medical students (N = 296) from 28 UK institutions responded to a 15-question online survey distributed via course leads and social media. Thematic analysis of qualitative data was conducted, as well as statistical analysis of quantitative data using SPSS.

**RESULTS:**

Only 40.9% of students reported having any teaching on LGBTQ+ healthcare, 96.6% of whom said this was one-off or very irregular sessions. Only 1 in 8 felt their knowledge and skills on LGBTQ+ healthcare was sufficient. 97.2% of students questioned wanted more knowledge on LGBTQ+ healthcare.

**CONCLUSION:**

The current study highlighted that UK medical students felt underprepared for working with LGBTQ+ patients due to insufficient education. Given that teaching on LGBTQ+ healthcare is often optional and extra-curricular, it may not be reaching those who need it most. The authors are calling for the mandatory inclusion of LGBTQ+ healthcare in the teaching of all UK medical schools, within their individual curriculum frameworks, and with regulatory support from the General Medical Council. This will ensure a wider understanding among medical students, and subsequently qualified doctors, of the health inequities and unique health issues LGBTQ+ people face, which will better equip them to provide high-quality care to LGBTQ+ patients, and start to tackle the inequities they face.

## Introduction

A key part of medical education is constant evolution. As medical knowledge develops and is built upon by novel research, it is important to continually evaluate the current medical curriculums and assess whether they are sufficiently preparing medical students for clinical practice. One area of medical curriculums that is often neglected is LGBTQ+ (lesbian, gay, bisexual, trans*, queer, questioning+) healthcare.^1^ Emerging research has started to reveal that UK medical curriculums are severely lacking in teaching on LGBTQ+ healthcare.^1–8^ However, existing studies exploring student experience have focused on single medical schools, making it difficult to understand the wider perspectives across the UK medical undergraduate system. The one study which has examined multiple medical schools (N = 19) in the United Kingdom, did so from the perspective of course leads, so does not reveal the views of medical students on their education.^1^ Although the literature does not provide a full picture of the extent, it is clear that teaching on LGBTQ+ healthcare is lacking in both quantity and quality.

When exploring the current teaching on LGBTQ+ healthcare in UK medical schools, the teaching currently largely conflates LGBTQ+ health with sexual health, leading to a dangerous hypersexualisation of the LGBTQ+ community, the perpetuation of inaccurate stereotypes and normative attitudes, as well as a lack of awareness of how LGBTQ+ identities intersect with health beyond sexual behaviour, which all impact on quality of care.^9–12^ While it is important to learn from past sexual health crises that significantly impacted the LGBTQ+ community, such as the 1980s AIDS crisis, and how the legacy of this still impacts how LGBTQ+ people engage with healthcare services,^13,14^ it is vital that the teaching of LGBTQ+ health extends beyond sexual health. The hypersexualisation of LGBTQ+ people, and particularly people who identify as queer, is dangerous due to the further stigmatisation of LGBTQ+ people that it can lead to. This can be associated with, and reinforce, stereotypes of queer people as sexually deviant, leading to the social isolation of LGBTQ+ communities.^15^

In addition to failings in the content of LGBTQ+ healthcare teaching, current literature has reported failings in the structure of the existing LGBTQ+ healthcare teaching in UK medical schools—specifically the vast variations seen between medical schools. Teaching on LGBTQ+ health is often in single modules, is in most cases not compulsory or assessed, and presents with inconsistencies between the amount and type of teaching that different medical schools provide on LGBTQ+ healthcare, with the amounts of teaching on LGBTQ+ healthcare ranging from 3 to 55 hours in the medical schools where it is taught.^1^

Sufficient teaching on LGBTQ+ health is key due to the known poorer health outcomes of the LGBTQ+ population: it is known that LGBTQ+ people have higher rates of poor mental and physical health.^16–19^ Further worsening this situation, they also experience greater levels of discrimination than their cisgender heterosexual counterparts, which can have negative impacts on accessing care.^16,17^ For example, lesbian and bisexual women are twice as likely to have never attended a cervical smear test than women overall, one reason for which may be fear of discrimination.^20^ This fear can also be further compounded by an element in some cases of lack of knowledge among healthcare professionals contributing to barriers to equal care. Over 20.0% of lesbian and bisexual women surveyed by Hunt and Fish^20^ had been told by a healthcare professional that they were not at risk of cervical cancer and did not require screening. In addition to fears of discrimination and lack of necessary knowledge, there are other barriers to LGBTQ+ people accessing healthcare: fear of being outed by mental health service providers,^21,22^ misgendering or inappropriate levels of curiosity in fertility and midwifery services,^23^ and policies limiting LGBTQ+ partners involvement in decision making during palliative care.^24^

These barriers are exacerbated when individuals lack confidence in their doctor's knowledge on LGBTQ+ health,^25^ therefore educating future doctors is a way to reduce these barriers. Reducing these barriers is key as barriers to accessing care may have significant impacts on public health outcomes and exacerbate health inequalities in LGBTQ+ populations.^9^ A key element of this is ensuring medical students are equipped sufficiently as a lack of training can have negative impacts on the quality of care that patients receive. This is clearly demonstrated in other areas of equality, diversity and inclusion (EDI) education such as race inequalities,^26^ disability teaching,^27^ and cultural competency.^28^ Thus, it is vital that the current failures in LGBTQ+ healthcare teaching are addressed to reduce barriers to care, and to improve the quality of care that LGBTQ+ patients are receiving.

It is, however, also important to acknowledge the barriers to including LGBTQ+ healthcare teaching in UK undergraduate medical curriculums, as this offers a potential explanation for the inconsistency in teaching and more importantly highlights what needs to be addressed to facilitate future change. One of the primary barriers cited in the literature is space and time in the curriculum—LGBTQ+ healthcare must compete for time and space with other areas of medicine and EDI education.^29^ Further barriers identified are lack of resources; lack of suitable knowledge; and not having staff that perceive themselves as qualified to teach LGBTQ+ healthcare.^30,31^

Likely in part due to these barriers, to date there has been no large-scale attempts at changing the medical undergraduate curriculum at governance level to improve LGBTQ+ healthcare teaching across all UK medical schools. Although some teaching resources have been produced, Hunt et al^31^ found that the current resources, or use of the resources, are still insufficient at equipping medical students to treat LGBTQ+ patients since discrimination is still seen against LGBTQ+ people in healthcare settings. There have previously been attempts to improve LGBTQ+ healthcare teaching in the UK medical schools, although these have mainly been on a small scale at single medical schools, and usually only with small groups of students, using methods such as self-assessment tools^32^; optional training courses^6^; experiential group work^33^ and 1-day teaching sessions. ^34,35^ Although some methods, such as experiential group work, have been effective,^33^ the literature overwhelmingly showed that current methods are not sufficiently equipping students.^31^ Existing methods are not sufficient due to allowing for opt-in bias^2,5^; not being consistent throughout curriculums and across medical schools^3,8^ and not assessing to ensure that changes have occurred in behaviour.^31^ Exploration of how LGBTQ+ healthcare education could be meaningfully included in the curriculum highlight practical methods of learning such as role play, communication skills workshops, clinical clerkships and focus groups with LGBTQ+ people,^4,8,35,36^ as well as theory-based learning methods such as essays, reflections, videos, presentations and expert panels.^3,8^

The current study aimed to ascertain a comprehensive picture of the current level of teaching on LGBTQ+ healthcare across UK medical schools; how well this was equipping medical students to treat LGBTQ+ patients; and the appetite among UK medical students for increased LGBTQ+ healthcare teaching in their curriculums. The research also aimed to provide a strong foundation for future curriculum development and amplify the voices of students asking for teaching on LGBTQ+ healthcare.

## Methods

### Design

This cross-sectional study employed a 15-question online questionnaire to explore the perceptions and training experiences of UK medical students in relation to LGBTQ+ health education. It elicited both quantitative and qualitative data. Ethical approval was obtained from the School of Medicine Research Ethics Committee at the University of Leeds (MREC 20-068).

### Participants and Sampling

An opportunity sample of medical students (N = 296) was recruited from 28 UK medical education institutions. This included medical students from all years of medical school, as well as students currently taking time out of medical studies (intercalating students). Students taking time out of studies were included as they have experienced teaching at medical school. All participants were asked to confirm they were above the age of 18 years. We did not exclude participants who did not complete the whole survey. This is due to some of the questions on the survey being optional, and some participants choosing not to respond to the open text questions. Respondents were asked to provide written consent by responding to a consent statement, and to confirm that they were medical students at a British medical school before completing the survey. The inclusion criteria were: being a current medical student, or student taking time out of their medical studies, at a UK medical school; being over the age of 18 years. The exclusion criteria were: not being a medical student; and not studying medicine in the United Kingdom. Responses were manually checked to prevent duplicate responses being included.

### Measures

The 15-question online survey was developed in association with LGBTQ+ individuals and medical students from the University of Leeds. This involved asking LGBTQ+ medical students for feedback on both the content and wording of the questionnaire. The questionnaire was designed to ascertain what education medical students in the United Kingdom were currently receiving on LGBTQ+ healthcare in their medical curriculums; how this impacted on their confidence in interacting with and treating LGBTQ+ patients; and what medical students wanted LGBTQ+ healthcare teaching to look like in their curriculum. Specific questions were asked looking at medical students’ knowledge of gender affirming care: this was in order to ascertain whether the training medical students were receiving addressed practical aspects of LGBTQ+ healthcare, as well as addressing attitudes and preconceptions surrounding LGBTQ+ patients. Students were asked only about one aspect of gender affirming care rather than about gender affirming care in general due to the wide breadth of what is contained within gender affirming care. The questionnaire used limited survey response options, rather than the Likert scale, in order to simplify the process of responding for students. The survey was pretested twice on volunteers whose feedback was used to improve wording and clarity of the questions. The full survey can be seen in Appendix 1.

### Procedure

The survey was uploaded to Jisc Online Surveys and subsequently advertised via social media platforms (Twitter, Instagram and LinkedIn), and sent directly to lead medical school faculty at all registered medical schools in the United Kingdom via email. On clicking the provided link participants were presented with the participant information sheet and online consent question; consent was necessary for the participant to gain access to the questionnaire. Participants were free to withdraw from the study at any point during completion of the questionnaire by closing the browser, however it was made clear during the consent process that due to the anonymous nature of the data collected it was impossible to identify and remove any data after submission. The survey was open for responses for a period of 6 weeks.

### Data Analysis

Quantitative data was analysed using SPSS. Descriptive statistics and the Kruskal–Wallis and Mann–Whitney U tests of difference were used. These were selected due to the non-parametric nature of the dataset. Thematic analysis was conducted on the open text questions (questions 8, 9 and 15) using Braun and Clarke's “Big Q” method, which involved immersion within the dataset, coding of ideas and then grouping codes into themes.^37^ This inductive analysis was completed independently by 3 members of the research group (AB, EP and JB), each with unique experiences of medical education and LGBTQ+ healthcare, and results were then discussed. Assumptions made by each person were debated and considered from various perspectives, in order to maintain reflexivity. The Consensus-Based Checklist for Reporting of Survey Studies (CROSS) was followed to improve the reliability and value of the research.^38^

## Results

### Participant Demographics

In total 298 responses were recorded, however 2 of these did not meet the inclusion criteria therefore they were removed. Consequently, 296 responses were used in the analysis. All respondents were medical students studying at a UK university. Of these, 65.2% (N = 191) of participants attended a medical school in England, 23.2% (N = 69) attended a medical school in Scotland, 10.9% (N = 32) attended a medical school in Wales and 0.7% (N = 2) attended a medical school in Northern Ireland. Of the English medical schools, they were spread across the north (N = 106), midlands (N = 5), south (N = 63) and London (N = 16). Overall, 28 medical schools were represented in the responses. The medical schools which were represented can be seen in Table 1. There was a suitable spread across year of study: year 1 (N = 36), year 2 (N = 52), year 3 (N = 79), year 4 (N = 61), year 5 (N = 46) and intercalating (additional year of study while completing a medical degree, usually in between the 3rd and 4th years of medicine) (N = 22). There was an overrepresentation of those identifying their sexuality as non-heterosexual when compared with general population data: 58.4% (N = 170) of participants reported their sexual orientation as non-heterosexual (gay N = 32, lesbian N = 27, bisexual N = 78, pansexual N = 12, asexual N = 2, queer N = 11, other = 8), with 37.8% (N = 110) identifying as heterosexual. This is in comparison to the 8.0% of those aged 16 to 24 years who identified as LGB in the Office of National Statistics Annual Population Survey in 2020.^39^ Of those who reported their gender identity 86.6% of participants were cisgender (cisgender female N = 183, cisgender male N = 62) and 6.4% were trans* (trans male N = 4, non-binary N = 14). People who identified as a gender outside of these groups, or who did not define or disclose their gender identity, were grouped together in a separate category (N = 20).

**Table 1.** Medical schools represented in the survey responses.

Region	Medical schools represented
England North	University of Leeds
	Newcastle University
	University of Sheffield
	Hull York Medical School
	University of Liverpool
England Midlands	University of Warwick
	University of Nottingham
	University of Birmingham
England South	Anglia Ruskin University
	University of Southampton
	University of Cambridge
	University of Oxford
	University of Bristol
	University of East Anglia
	University of Plymouth
London	King's College London
	Imperial College London
	St George's University London
	Queen Mary University of London
	University College London
Scotland	University of Edinburgh
	University of Glasgow
	University of Dundee
	University of Aberdeen
	University of St Andrew's
Wales	University of Cardiff
	University of Swansea
Northern Ireland	Queen's University Belfast

### Current LGBTQ+-Related Teaching in Medical Schools

**Figure 1.**
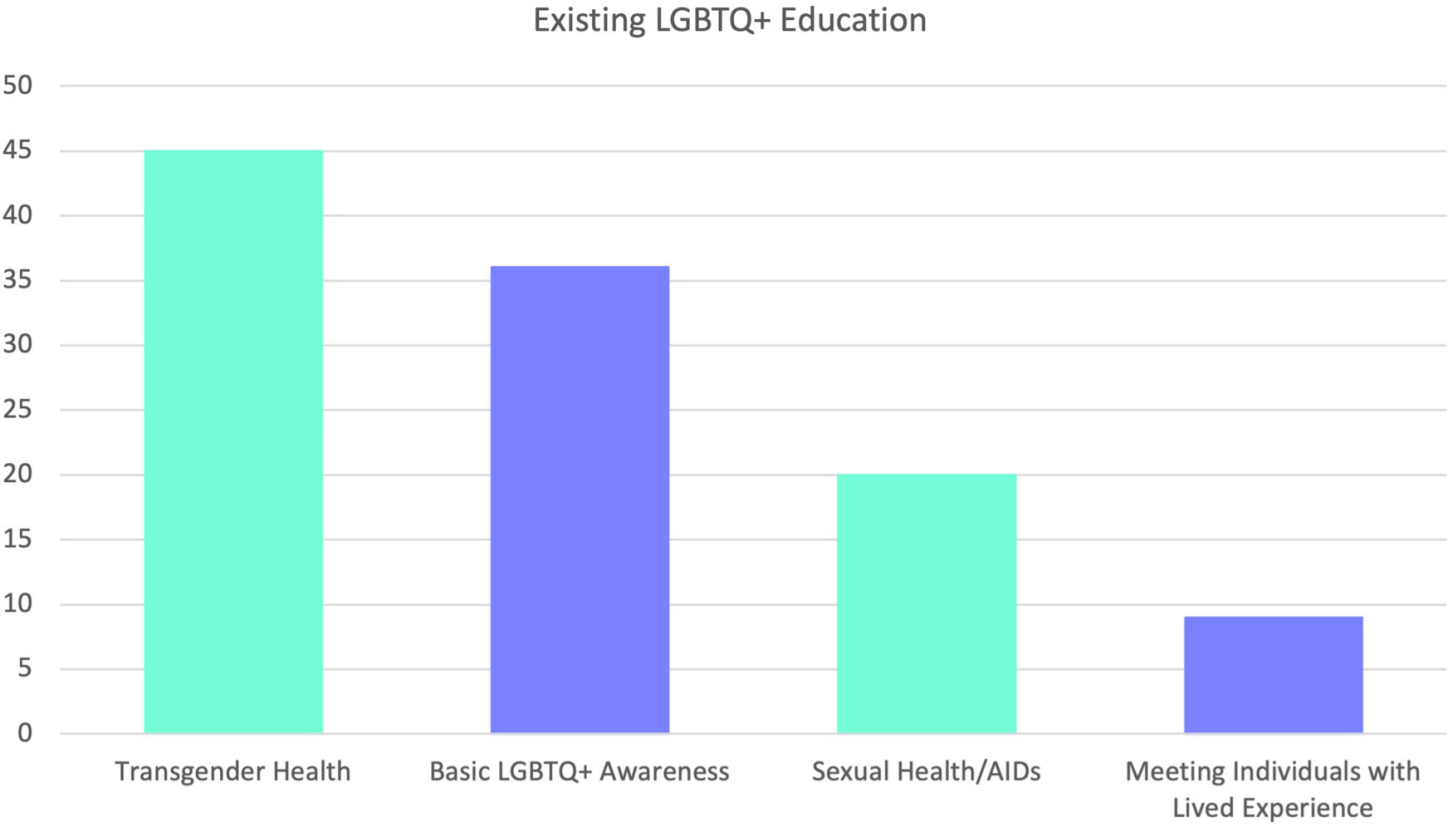
Content of teaching sessions received by those who stated they had received LGBTQ+ healthcare teaching. Abbreviation: LGBTQ+, lesbian, gay, bisexual, trans*, queer, questioning+.

51.4% (N = 147) of participants reported that they had not received teaching on LGBTQ+ healthcare, with a further 7.3% (N = 21) reporting they didn’t know. The 40.9% (N = 117) of participants who had received LGBTQ+ inclusive teaching were asked to elaborate on what this teaching involved. Thematic analysis of these responses generated 4 main themes regarding the content of this teaching, which is summarised in Figure 1.

Those who had received LGBTQ+ healthcare teaching were also asked to elaborate on the quantity of teaching they had received. Only 3.4% (N = 4) participants reported having regular teaching on the subject, with 95.0% reporting receiving a one-off session or irregular teaching. Many participants provided details on how these one-off sessions were not helpful due to all LGBTQ+ health content having to be fitted into rigid timeslots:

“It was very much a one day special, it wasn't woven into the curriculum and it felt very much as though the co-ordinator was only given this single day slot to fill all this teaching rather than integrating it throughout the curriculum.”

“It was one lecture in our second year of everything lgbtq shoved into a 45 min lecture. It was not helpful in any way.”

In addition to the limits created by one-off sessions, there were also some extreme examples given of how these sessions had been poorly handled. A 4th year student described poor teaching and the perpetuation of damaging stereotypes:

“I know past years’ have included LGBTQ+ info under ‘sexual deviation’ lectures which had info about paedophilia (unacceptable) and BDSM (kink shaming too). This year they said a slur during the lecture while listing out things NOT to say to trans patients. It was such a mess that [LGBTQ+ group] had to do [the] med school's work for them and teach, free of charge.”

While this example was not reflected widely and is likely an outlier, the extremity of this example warrants acknowledgment to prevent similar damaging teaching occurring in the future.

Analysis of exposure to LGBTQ+-related teaching across different demographics revealed two statistically significant findings. Firstly, the year of study significantly impacted if a student had received LGBTQ+ healthcare teaching (H(5) = 19.687, *P* = .001), with students in year 4 most likely to have received teaching (33 of 56 students) and students in year 1 (5 of 30 students) least likely: most likely due to the natural progression of students being taught about a wider breadth of subjects as they progress through medical school. Additionally, the medical school region significantly impacted if a student had received LGBTQ+ healthcare teaching. Medical students in London (9 of 13 students) were most likely to have received teaching, while Northern English (31 of 96 students) medical schools were least likely (H(6) = 14.422, *P* = .25).

### Knowledge and Understanding of LGBTQ+ Related Healthcare Issues

Participants were then asked to consider their knowledge and understanding of LGBTQ+ related healthcare issues. Firstly, they were asked to rate their level of understanding of gender affirmation surgery. Over half of participants reported “fully” (12.5%) or “partially” (53.0%) understanding the medical aspects of gender affirmation surgery. Participants who identified as LGB+ (74.0% N = 125) were significantly more likely to select either “fully” or “partially” understand when compared to those who identified as heterosexual (50.0% N = 54) (U = 6312.500, *P* < .001). Additionally, trans* participants (78.0% N = 14) were significantly more likely to select either “fully” or “partially” understand when compared to cisgender participants (62.0% N = 151) (U = 1549.00, *P* = .024). Nearly all participants (92.0%) reported having a “clear” understanding of the difference between sex and gender. Participants who identified as LGB+ (96.0% N = 162) were significantly more likely to report having a “clear” understanding when compared to participants who identified as heterosexual (86.0% N = 93) (U = 8124.000, *P* = .001).

Only 12.5% (N = 36) of participants felt their knowledge on LGBTQ+ healthcare was sufficient. The year of study significantly impacted if participants felt they had sufficient knowledge, year 5 students (12 of 15 students) were most likely to feel they have sufficient knowledge while intercalating students (1 of 7 students) were least likely to feel they have sufficient knowledge (H(5) = 14.440, *P* = .013). Additionally, participants who identified as LGB+ (15.5% N = 26) or trans* (39.0% N = 7) were significantly more likely to feel they had sufficient knowledge when compared to those who identified as heterosexual (7.0% N = 7) or cisgender (10.0% N = 33) (U = 6823.500, *P* < .001; U = 1466.000, *P* = .002).

Only 25.3% of participants reported they felt confident in treating LGBTQ+ patients when they qualified. The year of study significantly impacted students’ level of confidence treating LGBTQ+ patients after qualification, year 5 students (19 of 21 students) were most likely to select “yes” when asked if they felt confident, and year 3 students (14 of 23 students) were least likely to select “yes” (H(5) = 12.070, *P* = .034): this is potentially due to students' confidence generally growing as they progress through medical school. Participants who identified as LGB+ (31.0% N = 52) were significantly more likely to select “yes” than those who identified as heterosexual (14.0% N = 15) (U = 6352.500, *P* < .001). Participants who identified as trans* (47.0% N = 8) were significantly more likely to select “yes” than those who identified as cisgender (22.0% N = 53) (U = 1458.00, *P* = .022).

### Perspectives on the Need for LGBTQ+ Teaching at Medical School

Overall97.2% of participants said they would like more teaching on LGBTQ+ healthcare at medical school. Participants were asked to comment on how more teaching on LGBTQ+ healthcare would benefit their future practice as doctors. Thematic analysis of these responses generated several themes regarding how this teaching might benefit participants’ future practice, which is summarised in Table 2.

**Table 2.** Student responses to how they think teaching on LGBTQ+ healthcare would benefit their future practice.

Theme	N	Quotes
Develop their ability to meet patients’ needs	74	“I would be able to care for patients more appropriately and more holistically. I would feel safer treating them and more trained when approaching them.” “I would be able to provide better care for LGBTQ+ patients and have a better understanding of what support they need.” “… I would understand how to better care for LGBTQ+ patients so that their treatment could be as equally patient-centred and caring as it would be for a non-LGBTQ+ patient. …”
Develop their ability to communicate sensitively and respectfully	72	“Overall, it would allow consultations with LGBTQ+ individuals to be a more comfortable experience for both parties. It would also aid me in being more confident with using correct terminology and approaching conversations in the right way (eg, best way to ask patients their pronouns at the start of history taking).” “When teaching history taking there should be a devoted section to asking questions of a sexual or gender dysmorphia origin to make future doctors more comfortable broaching this topic.”
Develop their understanding of, and confidence in, LGBTQ+ specific health issues	67	“It would help me to better understand and empathise more with the struggles and stigma that the community faces.” “I would feel confident to discuss LGBT specific issues and have the vocabulary and understanding to engage productively in it. Furthermore, patients would feel more able to share information with me as a clinician if they feel confident that I understand what they are talking about.” “I would like to learn more about the issues and barriers LGBTQ+ people specifically may face, as this will improve my quality of care to patients through helping them to overcome the barriers. If we are not taught to identify them, they may go unresolved.”
Develop their understanding of LGBTQ+ experiences when accessing healthcare	45	“I think more people need to be aware of how to treat LGBTQ+ patients, to prevent healthcare avoidance due to fear of discrimination.” “We would definitely benefit from more inclusive teaching. There needs to be more focus on marginalised groups and their experiences within healthcare, how are we supposed to improve health outcomes for the most vulnerable if the education doesn't even begin in medical schools?”
Ensure that all students have sufficient knowledge on LGBTQ+ health provided from central-funded teaching, rather than the burden being on LGBTQ+ students with the self-gained knowledge to share their knowledge	19	“More teaching means that any cisgender and heterosexual allies don’t have to keep asking their queer counterparts for explanations.” “I feel I have understanding due to personal identity and significant personal study but feel most medical students are either incredibly under equipped at best or actively discriminative at worst.” “As part of the community I've educated myself on a lot of topics, so feel confident in such areas, but my colleagues who wouldn't have researched it wouldn't and that frightens me as it has bad consequences for the patients in terms of what contraception is allowed, screening, etc LGBTQ+ medicine is often seen as a debate, it's not up to debate, it should be presented as fact to students so they can practise safe, current medicine.”

Abbreviation: LGBTQ+, lesbian, gay, bisexual, trans*, queer, questioning+.

## Discussion

### The Current State of Teaching of LGBTQ+ Healthcare in UK Medical Curriculums

The current study supports the assertion of a severe lack of sufficient, sustained teaching on LGBTQ+ healthcare in UK medical schools that is presented in the current literature.^1,2,4^ This study builds further on the existing knowledge of single site studies^1^ by demonstrating a similar effect across multiple sites, UK wide. This therefore indicates a widespread issue, rather than an issue confined to individual medical schools.

This study found that 95.0% of participants had received either irregular, or a one-off, teaching sessions on LGBTQ+ related healthcare issues. This is firstly concerning since research in the area of primary and secondary education has shown that teaching on LGBTQ+ issues is ineffective in one-off sessions, and needs to be “infused throughout…education programmes.”^40^ Therefore, the current one-off sessions are likely insufficient to equip medical students. Further, in the past, EDI teaching in medical schools was treated as a minimal requirement to achieve compliance rather than to elicit real and lasting change, thus it is concerning that the current prevalence of one-off sessions is for this purpose only.^41^ We propose that future change on LGBTQ+ related teaching should move away from a compliance approach toward a truly inclusive curriculum focused on incorporating LGBTQ+ healthcare teaching consistently throughout the curriculum, rather than being delivered in a single session, in order to allow for a positive and meaningful impact on student learning and understanding.

In addition to concerns around the amount and consistency of teaching, there are also concerns about the content of the current teaching on LGBTQ+ health. Several participants (N = 20) stated that LGBTQ+ related teaching in medical curriculums was often limited to the area of sexual health, which supports existing findings.^1,5^ This approach risks influencing medical students’ perceptions of LGBTQ+ healthcare by furthering the hypersexualised stereotype of LGBTQ+ people.^7,42^ Such a simplification of LGBTQ+ people is dangerous for many reasons. Not only does it reinforce inaccurate societal stereotypes, but it overshadows the many other health concerns that LGBTQ+ people may present with. This may contribute to the aforementioned barriers to accessing healthcare which exacerbate the higher rates of poor mental and physical health.^16,17^ There is therefore a need to ensure that future change to the curriculum on LGBTQ+ healthcare is not only within sexual health teaching, but in all aspects of medical education. It is also key that the specific needs of trans* and non-binary patients are taught, as these can be different to those of LGB+ patients.

### The Impact on Patients and Public Health of the Current Lack of Teaching on LGBTQ+ Healthcare

The inadequacy of the current teaching on LGBTQ+ healthcare in medical curriculums to equip medical students to effectively treat LGBTQ+ people is further evidenced by the worrying finding that only 25.3% of our research participants reported feeling confident in working with LGBTQ+ patients upon qualifying. LGBTQ+ healthcare must be seen as equally important as other medical knowledge in order to ensure high quality of care for all patients. Just as work is being done in other areas to make healthcare inclusive of all patients, such as race equality,^43,44^ significant work is required for LGBTQ+ healthcare to be appropriately prioritised in medical education. Considering LGBTQ+ people have higher rates of poor mental and physical health and face greater levels of discrimination in the healthcare setting than their cisgender heterosexual counterparts,^16,17^ and doctors in all specialties will encounter LGBTQ+ patients, it is vital that medical students and clinicians have sufficient knowledge and skills in LGBTQ+ healthcare to be able to tackle these health inequalities.

As three-quarters of medical students did not feel confident to work with LGBTQ+ patients, it could be suggested that there may be a similar trend seen in doctors and qualified healthcare professionals. This is reflected in one Australian study which found that among healthcare professionals providing cancer care, many of them reported a lack of confidence in treating LGBTQ+ patients, stemming primarily from a lack of knowledge.^45^ This lack of knowledge and subsequent confidence may be a contributory factor towards the barriers to care, and sometimes discrimination, that LGBTQ+ patients currently face. This was reflected in two of the themes of the qualitative analysis found in this study; that more teaching on LGBTQ+ health would allow students to communicate with LGBTQ+ patients better, and that it would develop their understanding of and confidence in LGBTQ+-specific issues. One student stated that more teaching on LGBTQ+ health would allow them to “feel confident to discuss LGBT-specific issues and have the vocabulary and understanding to engage productively in it,” which reflects how knowledge can empower students and clinicians to communicate with and care for LGBTQ+ people better. While some may argue that a patient's sexuality or gender identity will not impact their care in specialties such as cardiology or neurology, there are nuances in communication and management decisions with LGBTQ+ patients that can greatly impact their care. For example, if patients experience discrimination or poor care in healthcare settings, they may be less likely to access care in the future and may experience poorer health outcomes overall.^9^

Additionally, there are LGBTQ+-specific health needs that medical students and clinicians need to be aware of. For example, as trans* and non-binary people may take hormone therapy, decisions regarding medication prescribing need to fully consider the wider patient needs, due to potential interactions and side effect profiles.^46^ In terms of drug interactions, it has been seen in some cases that the use of oestradiol can potentially reduce the plasma concentrations of PrEP (pre-exposure prophylaxis), therefore increasing the risk of HIV (human immunodeficiency virus) in trans* and non-binary people taking oestradiol.^47^ As well as drug interactions, it is also key to be aware of side-effect profiles of medications. An important example of this is the co-prescribing of antipsychotics, such as olanzapine, with testosterone. Olanzapine has been shown to increase total blood cholesterol levels,^48^ and testosterone use in trans* and non-binary people has been seen to increase blood pressure.^49^ Therefore, their combined use can increase the cardiovascular risk. This example again highlights that it is key that there is trans* healthcare-specific teaching, in order to meet the specific needs of trans* and gender diverse patients. These cases highlight the importance of increasing levels of specific LGBTQ+ healthcare teaching for medical students, and potentially for qualified healthcare professionals as well, to ensure a suitable level of care and improve the public health outcomes for the LGBTQ+ population in the United Kingdom. The desire for this teaching on LGBTQ+ specific health needs was also reflected as a key theme in the qualitative data of this study, with students making statements such as that teaching on LGBTQ+-specific health needs would allow them to be “aware of specific issues that might be unique to this [LGBTQ+ ] community.”

### Why is it Important that LGBTQ+ Healthcare is Taught to All Medical Students?

It is key that LGBTQ+ healthcare is taught to all medical students, and retained by all medical students, to ensure that all medical school graduates have the necessary knowledge on LGBTQ+ health. One potential way to achieve this is by including LGBTQ+ healthcare in assessments. Only two participants reported having LGBTQ+ healthcare included in their assessments at medical school. Research has shown that students’ learning is often dependent on what is likely to be assessed, and students are less likely to dedicate time to learning about topics that are not assessed, due to the high volume of examinable content that is already taught.^50,51^ Teaching on LGBTQ+ healthcare therefore needs to be assessed to ensure sufficient knowledge acquisition and behaviour change to have a positive impact on patient care.^31^ Including LGBTQ+ healthcare topics in assessments, for example, discussing cervical cancer screening with trans* and non-binary patients, or situations including sensitively asking a patient about their internal reproductive organs, is likely to further encourage students to engage with teaching and revision on these topics.

Additionally, mandating assessment on LGBTQ+ healthcare will ensure that all medical students leave medical school with the same minimum level of understanding of LGBTQ+ health. This is especially important since the current study found heterosexual and cisgender participants had significantly lower levels of knowledge than there LGB+ and trans* counterparts. Despite this finding, current literature suggests that it is still LGBTQ+ students that are more likely to attend optional training on LGBTQ+ healthcare than non-LGBTQ+ students. ^5,6^ Therefore, assessing content could counteract the opt-in bias that is seen in optional training. While assessment is not necessarily the only way, or the best way, to ensure that all students actively engage with teaching and learning on LGBTQ+ healthcare, it is an established way, which medical schools will be familiar with, to ensure that medical students have the necessary knowledge on LGBTQ+ healthcare when they graduate.

Additionally, this study found that LGBTQ+ students felt that their personal experience and self-taught knowledge on LGBTQ+ health, which they may share with peers, was not an appropriate substitute for taught content on LGBTQ+ health for all students. Thus, universal teaching for all students is necessary. One participant said, “As part of the community I've educated myself on a lot of topics, so feel confident in such areas, but my colleagues who wouldn't have researched it wouldn't and that frightens me as it has bad consequences.” This reflects that the personal experience and expertise of LGBTQ+ medical students cannot be relied upon to equip the whole future medical workforce. Although personal experiences of LGBTQ+ healthcare can allow clinicians to better empathise with their patients, it is still necessary for those without personal experience of being LGBTQ+ to feel confident in managing LGBTQ+ patients’ health needs which requires teaching for all students on LGBTQ+ health.

Students also felt that LGBTQ+ medical students had to supplement their classmates’ learning with their own personal experiences. One participant said that “More teaching [would] means that any cisgender and heterosexual allies don’t have to keep asking their queer counterparts for explanations”; which reflects the burden that is currently placed on many LGBTQ+ medical students. This alone cannot be a substitute for taught content from subject experts as it puts burden on the minoritised students, rather than the curriculum. It is important to move away from the expectation that those in minority groups have the responsibility to educate their peers as this furthers stress experienced by those who are already oppressed. Therefore, structured and assessed teaching is likely to ensure that the students who need the teaching will receive it and begin to remove the additional pressure on LGBTQ+ students to educate their peers.

### What Needs to Change and How Should This be Done?

The authors call for a centralised change by governing bodies to the required learning outcomes of UK medical graduates, to include regulated mandatory learning outcomes on LGBTQ+ healthcare. It is key that the General Medical Council (GMC) takes responsibility for mandating the teaching of LGBTQ+ healthcare, throughout UK medical schools, to ensure all medical students receive sufficient education. A key aspect of this is inclusion of the teaching of LGBTQ+ healthcare in future revisions of the GMC's “Outcomes for Graduates” framework.^52^ Mandated outcomes will ensure that all medical schools are required to teach LGBTQ+ healthcare, which will increase the likelihood of dedicated staff to be allocated to teaching LGBTQ+ health, rather than the burden being automatically placed on LGBTQ+ students and staff. It is also important that the Medical Schools Council (MSC) includes the assessment of LGBTQ+ healthcare in the Medical Licensing Assessment so that all medical students will have their knowledge of LGBTQ+ healthcare assessed.^53^

It is important to recognise that centralised change will take time, but smaller actions can also make meaningful change while larger systemic changes progress. Individual medical schools should be encouraged to increase their teaching on LGBTQ+ healthcare. This can be done by including LGBTQ+ patients in clinical scenarios, communication skills sessions and simulation training. By integrating LGBTQ+ patients and healthcare scenarios into existing teaching frameworks, medical schools can overcome some of the potential barriers identified by Tollemache et al^1^ such as lack of space in the timetable. As well as this allowing opportunities to introduce LGBTQ+ healthcare-specific issues, this will also allow for joyful representation of LGBTQ+ people. Participants of the current study also suggested the benefits of learning from people with lived experience of LGBTQ+ healthcare, such as talks from LGBTQ+ patients, with students making statements such as “a trans woman was a guest speaker for this session and spoke of her own experiences. This was hugely beneficial.” Personal stories have proven an effective teaching method in teaching about birth experiences in midwifery education^54^ and are often used in teaching medical students about patient experiences of chronic conditions or disabilities.^55^

There are also existing resources on LGBTQ+ health which medical schools can use or direct students to, even if they have potential limitations. For example, the Royal College of General Practitioners created an e-learning package on LGBTQ+ healthcare for general practitioners and healthcare professionals to use.^56^ E-learning packages are a good way to introduce students to the topic of LGBTQ+ healthcare, however, it is important to note that e-learning alone is insufficient to teach all that is required on LGBTQ+ healthcare. Teaching on LGBTQ+ healthcare should encourage students to reflect on their own practice and promote discussion about preconceived opinions and subconscious biases.^1^ In topics like this, which require discussion in class, e-learning is clearly insufficient.^57^ Implementation of a variety of teaching methods, addressing specific issues faced by both LGB+ and trans* and non-binary patients, is required to ensure future clinicians can approach LGBTQ+ healthcare in a holistic manner. A systemic change in the perceived importance of LGBTQ+ healthcare in the medical curriculum is required to foster an inclusive curriculum that will benefit LGBTQ+ patient outcomes.

### Strengths and Limitations

Regarding the strengths of this study, despite the relatively small sample size, this is the largest multisite study on LGBTQ+ healthcare teaching in UK medical schools to date. It does, however, shine light on the lack of research in this area and leads the researchers to advocate for funding for future larger studies in this area. The study also had respondents from all years of medical school and from all regions of the United Kingdom which increases its representation of medical curriculums across the UK There was also a significant number of respondents from the North of England which has historically been underrepresented in research on LGBTQ+ healthcare teaching in medical schools. Another strength of this study is the use of both quantitative and qualitative data. This allows for both an objective investigation of the number of medical students reporting insufficient teaching on LGBTQ+ health, as well as allowing for the individual perspectives of medical students from across the country to be explored.

There are also limitations to this study. Firstly, this is still a relatively small study considering the number of medical students in the United Kingdom. The sample size was not calculated due to the predicted potential difficulties in recruiting medical students, therefore, the sample instead aimed to include as many medical students as the study was able to reach. Additionally, there was an overrepresentation of LGBTQ+ participants and a very low percentage of respondents from certain geographical areas such as Northern Ireland. It is expected that LGBTQ+ individuals are more likely to engage in research related to LGBTQ+ health as they have a personal investment, but this also highlights a potential perception of a lack of importance in heterosexual cisgender individuals. The overrepresentation of LGBTQ+ individuals may lead to an inaccurate picture of how much knowledge students have on LGBTQ+ health, and how confident students are, as LGBTQ+ students may know more about LGBTQ+ issues due to their involvement in the LGBTQ+ community. LGBTQ+ people may also be more likely to think the current teaching on LGBTQ+ health is insufficient due to their personal investment and knowledge of issues in LGBTQ+ healthcare. Moreover, it is especially important to further investigate the teaching of LGBTQ+ health in each individual nation of the United Kingdom rather than assuming perceptions in England will generalise to the wider UK. For example the differing attitudes in Northern Ireland around LGBTQ+ rights may lead to teaching on LGBTQ+ health differing dramatically,^58^ therefore it is vital further understanding is gained. To conduct larger, more representative studies in the area of LGBTQ+ health teaching in UK medical schools, there is a need for funding into research, as well as support from governing bodies such as the GMC and MSC.

## Conclusion

To conclude, the current study demonstrated that nationally there is a lack of teaching on LGBTQ+ healthcare in UK medical schools curriculums, and this is resulting in medical students being insufficiently prepared to treat LGBTQ+ patients. The findings highlight a significant appetite, and need, among medical students to have more teaching on LGBTQ+ healthcare incorporated into medical curriculums. The authors call on the central governing bodies to mandate the teaching of LGBTQ+ healthcare in UK medical schools. The authors urge governing bodies to involve key LGBTQ+ stakeholders in these changes. The authors also urge individual medical schools to evaluate their own teaching, and to assess how they can increase the amount of teaching on LGBTQ+ healthcare in their curriculums.

## Appendix 1: Survey

Investigation into British medical students understanding of LGBTQ+ healthcare and the level of LGBTQ+ inclusive healthcare teaching in the UK undergraduate medical curriculum.

### Project information

Thank you for taking the time to read this information sheet.

LGBTQ+ (lesbian, gay, bisexual, transgender, queer and questioning +) patients face many barriers to accessing healthcare. One way that these barriers can be tackled is by educating future healthcare professionals to provide a more inclusive health service. In order to better understand the current curriculum and training needs around LGBTQ+ inclusive healthcare it is necessary to get a wider understanding of the perspectives and experiences of UK medical students. The current survey aims to collect the experiences and perspectives of UK medical students, to understand if their training translated into LGBTQ+ inclusive practise, and how the curriculum could be improved to inform future change to the UK medical curriculum to integrate teaching of LGBTQ+ inclusive healthcare.

### What is this survey?

It is an online survey consisting of 15 questions which are either multiple choice or free text answers. This should take you 10 min to complete. The questions are surrounding your understanding of LGBTQ+ healthcare, and how much teaching you feel you have had in this subject. The only personal questions are your year group, medical school, gender identity and sexual orientation—you can choose whether you answer these or not.

### What do I have to do?

You are invited to complete an online questionnaire which should take no more than 10 min to complete. This is completely anonymous.

### Do I have to do this?

Participation is completely voluntary. By choosing to submit the form at the end you are submitting your answers to be used in the study. You can withdraw consent at any point until you submit your answers by simply closing the browser window.

### Who is carrying out this research?

This research is being carried out by medical students at the University of Leeds, the project lead is Alice Barber (Medical Student at the University of Leeds) and being supervised by Dr Emily Pattinson (Research Fellow at the University of Newcastle).

Other researchers:

Jessica Burt (Medical Student of University of Leeds) Alex Flach (Medical Student of University of Leeds).

Jack Bonnington (Medical Student of University of Leeds).

### What are the possible benefits of taking part?

The survey will allow medical students to have your say on your education regarding LGBTQ+ healthcare. Which aims to inform changes to the curriculum and indirectly improve care for LBGTQ+ patients.

### What are the risks of taking part?

There is very minimal risk to taking part in this study and is unlikely to cause distress. The only questions which are potentially sensitive are inviting you to share your sexual orientation and gender identity. These answers will not be able to be linked to you. You also have the option to select “prefer not to say.”

### Can my answers be linked to me?

We are not collecting any identifiable information therefore all answers will remain anonymous and participation will be kept confidential. The only questions relating to you will be your medical school, year group, sexual orientation and gender identity, but there will be a “prefer not to say” option for all of these questions. Due to the fact that answers are anonymous, once you have submitted the answers you will not be able to withdraw from the study. The data will be stored in a password-protected file and stored in the University of Leeds cloud storage. If any quotations are used these will be kept anonymous.

### What will happen with the results of the research?

The data will be collated and a report will be written. This will then be submitted for journal publication and potentially presented at academic conferences. Direct quotes may be used anonymously, but no identifiable data will be presented or reported.

### Who has reviewed this study?

The study has been reviewed by the School of Medicine Research Ethics Committee on April 05, 23 and granted ethical approval: MREC 20-068

### Who can I contact if I have any questions?

Lead Investigator: Alice Barber um18a5b@leeds.ac.uk. Telephone: 07722265082

Project Supervisor: Dr Emily Pattinson emily.pattinson@newcastle.ac.uk

**Consent Statement:** By answering “YES” you consent to participating in this study, and you agree that you are over the age of 18 years and have read and understood the information you have been provided regarding the survey. You are also stating that you understand that since all responses are anonymous, they cannot be withdrawn once they are submitted. All information provided, including any direct quotations, will remain anonymous. Date: March 29, 2021 Version number 3 Ethics approval code: MREC 20-068 Contacts: Alice Barber um18a5b@leeds.ac.uk (Lead Investigator), Emily Pattinson emily.pattinson@newcastle.ac.uk (Project Supervisor) ⍰ *Required*

### Survey questions

Are you eligible to take part in this study (ie, a medical student at a university in the UK)?

⍰ Required

Which year group of medical school are you in?

Which UK medical school are you at?

What is your sexual orientation? *Optional*

What is your gender identity? *Optional*

Have you had any teaching at your medical school on how to make LGBTQ+ healthcare inclusive?

If you have had teaching on LGBTQ+ healthcare—what was included in this teaching?

Optional

If you have had teaching on LGBTQ+ healthcare—how much teaching have you had and was this regular teaching or a one-off session? *Optional*

Do you understand the medical aspects of gender confirmation surgery?

Do you know the difference between sex and gender?

Do you feel that your knowledge on LGBTQ+ inclusive healthcare is sufficient?

Do you feel confident in treating LGBTQ+ patients when you qualify?

Would you like more teaching on LGBTQ+ healthcare?

How do you think more teaching on LGBTQ+ healthcare would benefit your future practise? *Optional*

### Denied consent/not eligible

Thank you for taking the time to read this survey. You have been directed to this page either because you did not consent to take part in the survey or you are not eligible to take part in the survey. If this was out of error or you change your mind then please feel free to go back and complete the survey. Thank you again for your time.

If you have any further questions please contact:

Lead Investigator: Alice Barber um18a5b@leeds.ac.uk

Project Supervisor: Emily Pattinson emily.pattinson@newcastle.ac.uk

If you need further support and are a students at Leeds, please contact the student support team at Leeds: https://medicinehealth.leeds.ac.uk/medicine-undergraduate/doc/student-support-1. If you are a student elsewhere please contact your respective student support team.

Support can also be found at https://switchboard.lgbt or https://www.kooth.com irrespective of university of study.

### Final page

Thank you for taking the time to complete this survey today, your time and responses are appreciated. All answers that you provided will remain completely anonymous.

If you have any further questions please contact:

Lead Investigator: Alice Barber um18a5b@leeds.ac.uk

Project Supervisor: Emily Pattinson emily.pattinson@newcastle.ac.uk

If you need further support and are a students at Leeds, please contact the student support team at Leeds: https://medicinehealth.leeds.ac.uk/medicine- undergraduate/doc/student-support-1. If you are a student elsewhere please contact your respective student support team.

Support can also be found at https://switchboard.lgbt or https://www.kooth.com irrespective of university of study.
